# Tracking spatiotemporal distribution of organelle contacts in vivo with SPLICS reporters

**DOI:** 10.1038/s41419-025-07511-5

**Published:** 2025-03-27

**Authors:** Lucia Barazzuol, Tetiana Tykhonenko, Tia L. Griffiths, Alessio Vagnoni, Marisa Brini, Tito Calì

**Affiliations:** 1 Department of Biomedical Sciences (DSB), University of Padova, Padova, Italy; 2 Department of Basic and Clinical Neurosciences, Maurice Wohl Clinical Neuroscience Institute, Institute of Psychiatry, Psychology and Neuroscience, King’s College London, London, UK; 3 Multidisciplinary Institute of Ageing, University of Coimbra, Coimbra, Portugal; 4 Department of Pharmaceutical and Pharmacological Sciences, University of Padova, Padova, Italy; 5 Study Center for Neurodegeneration (CESNE), University of Padova, Padova, Italy; 6 Padova Neuroscience Center (PNC), University of Padova, Padova, Italy

**Keywords:** Proteins, Diseases

## Abstract

Organelle contact sites are crucial for cellular function, enabling the exchange of lipids, ions, and other molecules between different organelles. The ability to track these contact sites in vivo has been significantly advanced by the development of SPLICS (Split-GFP-based Contact Site Sensors) reporters, which have provided unprecedented insights into the intricate network of organelle communication. This innovative and powerful tool allows the real-time visualization of different organelle interactions in living cells and in vivo thus unraveling the complexity of their dynamic in the context of cellular homeostasis. Recent studies highlighted the dynamic nature of organelle contact sites either in terms of tethering/untethering and of movement of the contact itself in time and space: whether unique temporal behaviors and contact site-specific dynamics of different organelle interactions exist is currently unknown. In this study, we investigated the spatiotemporal distribution of various organelle contact sites using time-lapse in vitro and in vivo imaging and discovered an evolutionarily conserved dynamic pattern among different contact sites, influenced by the specific partner organelles involved. These findings highlight the importance of spatial and temporal regulation at organelle contact sites, which may underlie their diverse physiological functions. The discovery of contact site-specific dynamics opens new avenues for understanding the regulation of organelle interactions in health and disease, with potential implications for developing targeted therapeutic strategies.

## Introduction

Organelle contact sites play critical roles in intracellular signaling, the exchange of ions and molecules, lipid metabolism, membrane dynamics, organelle division, and biogenesis. Over the past decade, research in this field has gained significant momentum, leading to the widespread recognition that all organelles establish functional contacts with one another. The understanding of these contact sites is continually evolving, with ongoing discoveries revealing new roles and underlying mechanisms that govern these interactions. Cellular organelles are dynamic structures constantly undergoing fission/fusion and remodeling; therefore, a highly dynamic interaction scheme is also observed for organelle-organelle interactions [1]. So far, the number of studies specifically addressing the dynamic nature of organelle contact sites is very limited due to the scarce availability of high-resolution imaging techniques coupled to the lack of tools to image contact sites behavior in vitro and in vivo. By using SPLICS reporters [2–6] we have explored the dynamic nature of ER-mitochondria contacts by confocal microscopy both in vitro and in vivo [6]: long-range interactions were more extensively remodeled compared to short-range ones, which are characterized by a longer half-life, slower speed, and higher probability of movement. More recently, 3D electron microscopy with high-speed tracking of HaloTag-VAPB, revealed the existence of dynamic subdomains matching the ER-mitochondria contact sites some of which remained stable over 60–90 s of imaging [7]. ER-Plasma membrane contacts have also been successfully tracked over time [8], revealing their peculiar polarization within minutes. However, whether evolutionarily conserved contact-site specific dynamics exist is still unknown. To delve deeper into this question, we conducted time-resolved imaging experiments in vitro and in vivo, quantifying the kinetics of four different organelle contact sites using SPLICS reporters: SPLICS_S_-P2A^ER-MT^, SPLICS_S_-P2A^LY-MT^, SPLICS_S_-P2A^PO-MT^ and SPLICS_S_-P2A^ER-PM^ [5, 6]. To identify evolutionary conserved behaviors, we have i) expressed them in *Danio rerio* Rohon-Beard (RB) neurons, ii) generated transgenic *Drosophila melanogaster* flies expressing SPLICS in the wing sensory neurons iii) expressed them in mouse derived motor neuron cell lines. Different parameters have been quantified by time-resolved imaging experiments revealing an overall conserved dynamic behavior of organelle contacts, with the speeds ranking as follows: ER-PM > ER-MT = PO-MT > LY-MT, highlighting the importance of spatial and temporal regulation at organelle contact sites, which may underlie their diverse physiological functions and suggesting that SPLICS reporters can be valuable tools to study contact dynamics in vitro and in vivo.

## Results

### Expression and characterization of SPLICS_S_-P2A^ER-MT^, SPLICS_S_-P2A^ER-PM^, SPLICS_S_-P2A^PO-MT^ and SPLICS_S_-P2A^LY-MT^ in Danio rerio Rohon-Beard (RB) neurons

**Fig. 1:**
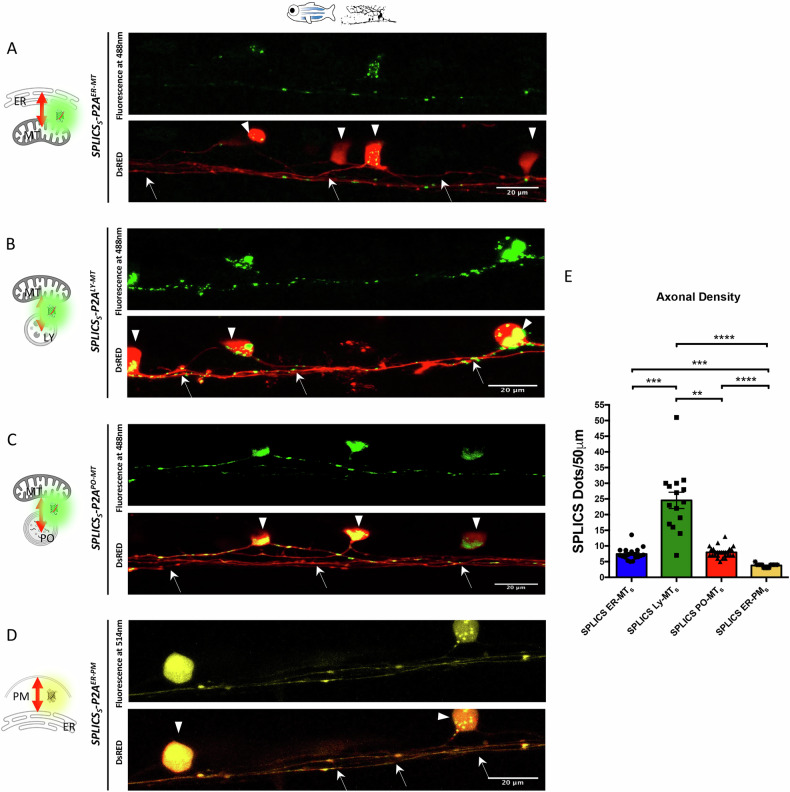
Expression of SPLICS in *Danio rerio* Rohon-Beard neurons. RB neurons expressing (**A**) *SPLICS*_*S*_*-P2A*^*ER-MT*^ (**B**) *SPLICS*_*S*_*-P2A*^*LY-MT*^ (**C**) *SPLICS*_*S*_*-P2A*^*PO-MT*^ and (**D**) *SPLICS*_*S*_*-P2A*^*ER-PM*^ along with a cytosolic DsRed. The typical punctate pattern of SPLICS is observed in the soma (arrowhead) and in the axons (arrows). Axonal density of contact sites is quantified in (**E**). Scale bar is 20 μm. N of axons= 18, 15, 27, 17. Statistical analysis was performed using Kruskal–Wallis test with Dunn’s multiple comparison. **P < 0.01, ***P < 0.001, ****P < ; 0.0001. Scale bar is 20 μm.

Building on the established applicability of the SPLICS system in *Danio rerio* [2, 4–6], we utilized this model organism to conduct focused analyses aimed at further characterizing the behavior of different organelle contacts in an in vivo setting. For this purpose, by using the GAL4/UAS system, we transiently and tissue-specifically expressed four SPLICS probes in zebrafish embryos, targeting ER-MT, ER-PM, PO-MT, and LY-MT short-range contacts, respectively. As described by Vallese et al. [6], we generated new constructs that allow the expression of these SPLICS probes, along with a cytosolic dsRED marker, specifically in a subpopulation of zebrafish mechanosensory neurons known as the Rohon-Beard (RB) neurons. Representative images (Fig. 1A–D) show that all tested SPLICS probes exhibited a dotted pattern when transiently expressed in RB neurons of 1 dpf embryos, consistent with expectations and further validating the in vivo applicability of the SPLICS system. Co-localization of mitochondria was also checked for mitochondria-involving contacts (Supplementary Fig. 1). Given that SPLICS quantification in the soma of RB neurons is difficult due to its small and densely packed environment, when not possible, we chose to quantify the number of contacts in the axons (arrows), which are less crowded and more defined compartments. This approach allowed for more precise and relatively easier quantification. Upon comparing the distribution of the different contacts within the first 50 µm of neuronal axons (Fig. 1E), we observed that each type of contact site has its own density, being the number of dots in the 50 µm different for each of them (Fig. 1A–D). These findings suggest that contact density is peculiar for each type of membrane contact site and that it is not influenced by the expression of the SPLICS system itself. In particular, LY-MT contacts were highly prevalent in RB axons, whereas ER-PM contacts were relatively rare. ER-MT and PO-MT contacts showed intermediate similar abundance (values Mean ± SEM: SPLICS_S_-P2A^ER-MT^ = 7.461 ± 0.4717, *N* = 18; SPLICS_S_-P2A^ER-PM^ = 3.765 ± 0.1364, *N* = 17; SPLICS_S_-P2A^PO-MT^ = 7.963 ± 0.3268, *N* = 27; SPLICS_S_-P2A^LY-MT^ = 24.53 ± 2.607, *N* = 14) but lower than the LY-MT contacts.

### Time-lapse imaging of SPLICS_S_-P2A^ER-MT^, SPLICS_S_-P2A^ER-PM^, SPLICS_S_-P2A^PO-MT^ and SPLICS_S_-P2A^LY-MT^ in Danio rerio Rohon-Beard (RB) neurons

**Fig. 2:**
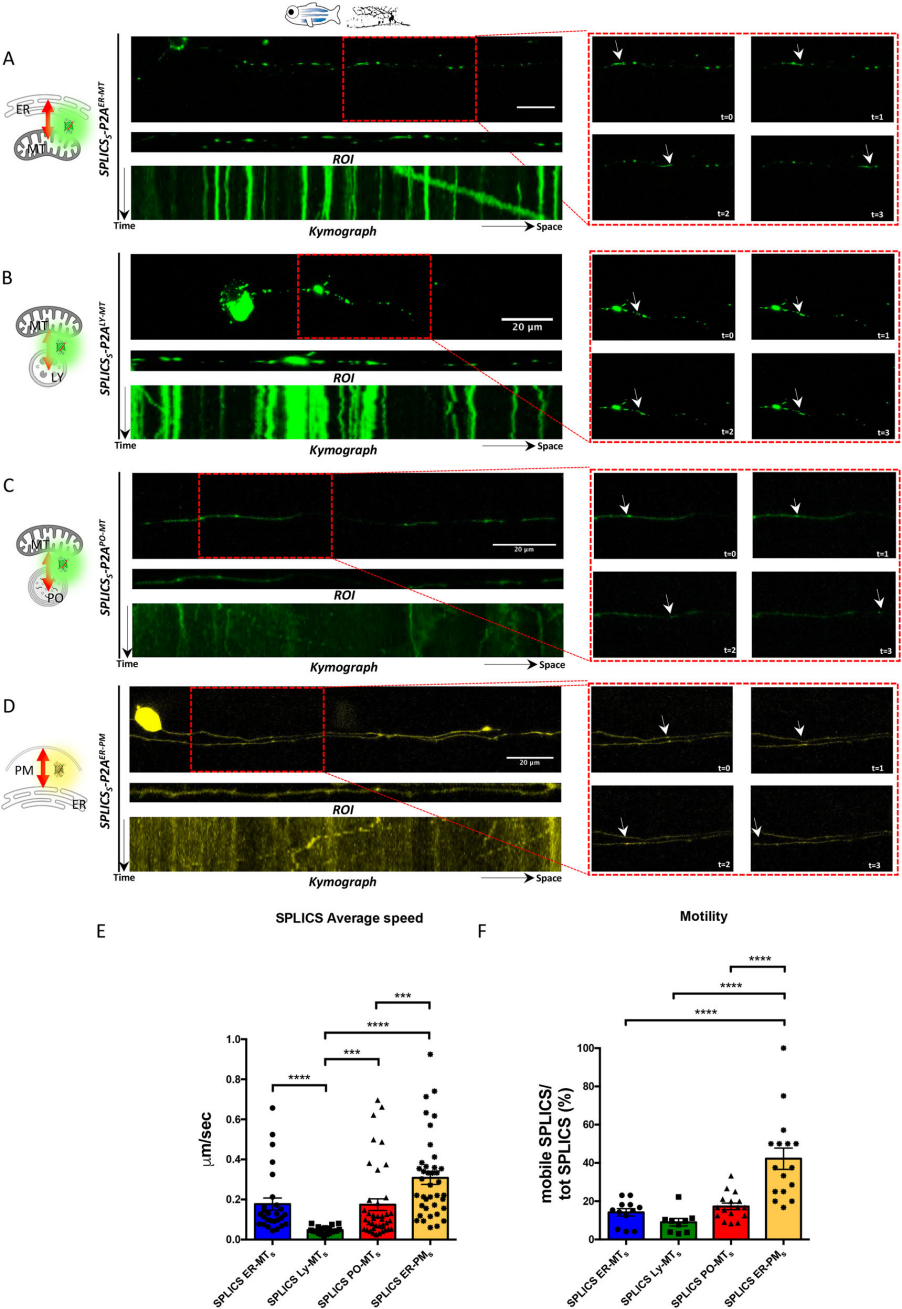
Time-lapse experiments of SPLICS in *Danio rerio* Rohon-Beard neurons. RB neurons expressing (**A**) *SPLICS*_*S*_*-P2A*^*ER-MT*^ (**B**) *SPLICS*_*S*_*-P2A*^*LY-MT*^ (**C**) *SPLICS*_*S*_*-P2A*^*PO-MT*^ and (**D**) *SPLICS*_*S*_*-P2A*^*ER-PM*^ were imaged over time, ROI of aligned neuronal axons and kymograph is shown. In the right panel a frame-by frame images of selected time points are shown (arrowhead indicates a specific contact site). **E** Average speed of SPLICS contact sites in μm/sec is quantified. N of moving dots= 27, 19, 40, 39. Statistical analysis was performed using Kruskal–Wallis test with Dunn’s multiple comparison. ***P < 0.001, ****P < 0.0001. **F** Quantification of the mobile SPLICS over the total SPLICS (%). N of axons= 12, 9, 16, 16. Statistical analysis was performed using one-way ANOVA and Tukey’s multiple comparisons. ****P < 0.0001. Scale bar is 20 μm.

Given the dynamic nature of these contacts, we conducted time-lapse experiments on RB neurons to better characterize contact behavior over time. Neurons, due to their distinctive architecture, serve as an excellent model for understanding the logic of intracellular trafficking. Axons, in particular, provide an optimal setting for investigating contact dynamics, as they offer a clearer and more manageable environment for tracking particle movements compared to the soma. When we imaged RB axons over time, the different contact sites displayed varying dynamics (Fig. 2A–D, and right panels), further supporting their specificity. This is highlighted by the kymograph analysis which shows a stalling contact as a straight line and a mobile contact as a dashed/oblique line. Specifically, LY-MT contacts exhibited low mobility (*SPLICS*_*S*_*-P2A*^*LY-MT*^ average speed = 0.04752 µm/sec; motility = 9.000%), while ER-PM contacts showed higher mobility (*SPLICS*_*S*_*-P2A*^*ER-PM*^ average speed = 0.3082 µm/sec; motility = 42.23%). ER-MT and PO-MT contacts showed intermediate and similar kinetics (*SPLICS*_*S*_*-P2A*^*ER-MT*^ average speed = 0.1773 µm/sec; motility = 14.16%; *SPLICS*_*S*_*-P2A*^*PO-MT*^ average speed = 0.1741 µm/sec; motility = 17.26%), suggesting that even when a common organelle is engaged (e.g., mitochondria) the contact with the partner organelle determines the dynamics of the contact site which can vary consistently. Supplementary Movies S1A–D are the raw original images.

### Generation and time-lapse imaging of SPLICS_S_-P2A^ER-MT^, SPLICS_S_-P2A^ER-PM^, SPLICS_S_-P2A^PO-MT^ and SPLICS_S_-P2A^LY-MT^ transgenic Drosophila melanogaster lines

**Fig. 3:**
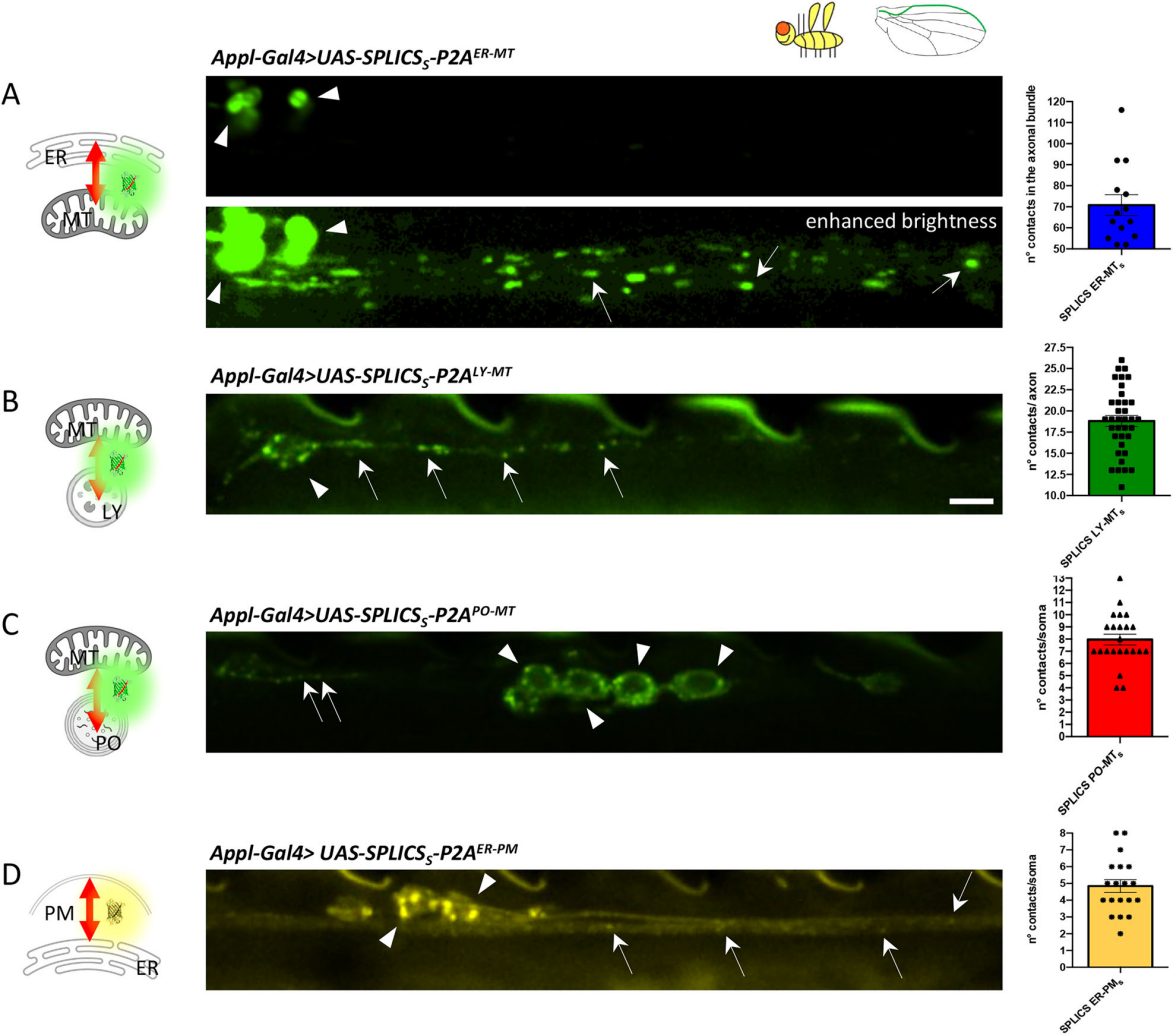
Expression of SPLICS in transgenic *Drosophila melanogaster* wing neurons. Wing neurons expressing (**A**) *UAS-SPLICS*_*S*_*-P2A*^*ER-MT*^ (**B**) *UAS-SPLICS*_*S*_*-P2A*^*LY-MT*^ (**C**) *UAS-SPLICS*_*S*_*-P2A*^*PO-MT*^, and (**D**) *UAS-SPLICS*_*S*_*-P2A*^*ER-PM*^ in the cell bodies and axons of the Drosophila wing. The typical punctate pattern of SPLICS is observed in the soma (arrowheads) and in the axons (arrows). Due to disparity of *SPLICS*_*S*_*-P2A*^*ER-MT*^ signal intensity between cell bodies and axons, in (**A**) the axonal contact sites are revealed after enhancing image brightness. Scale bar is 5 μm.

**Fig. 4:**
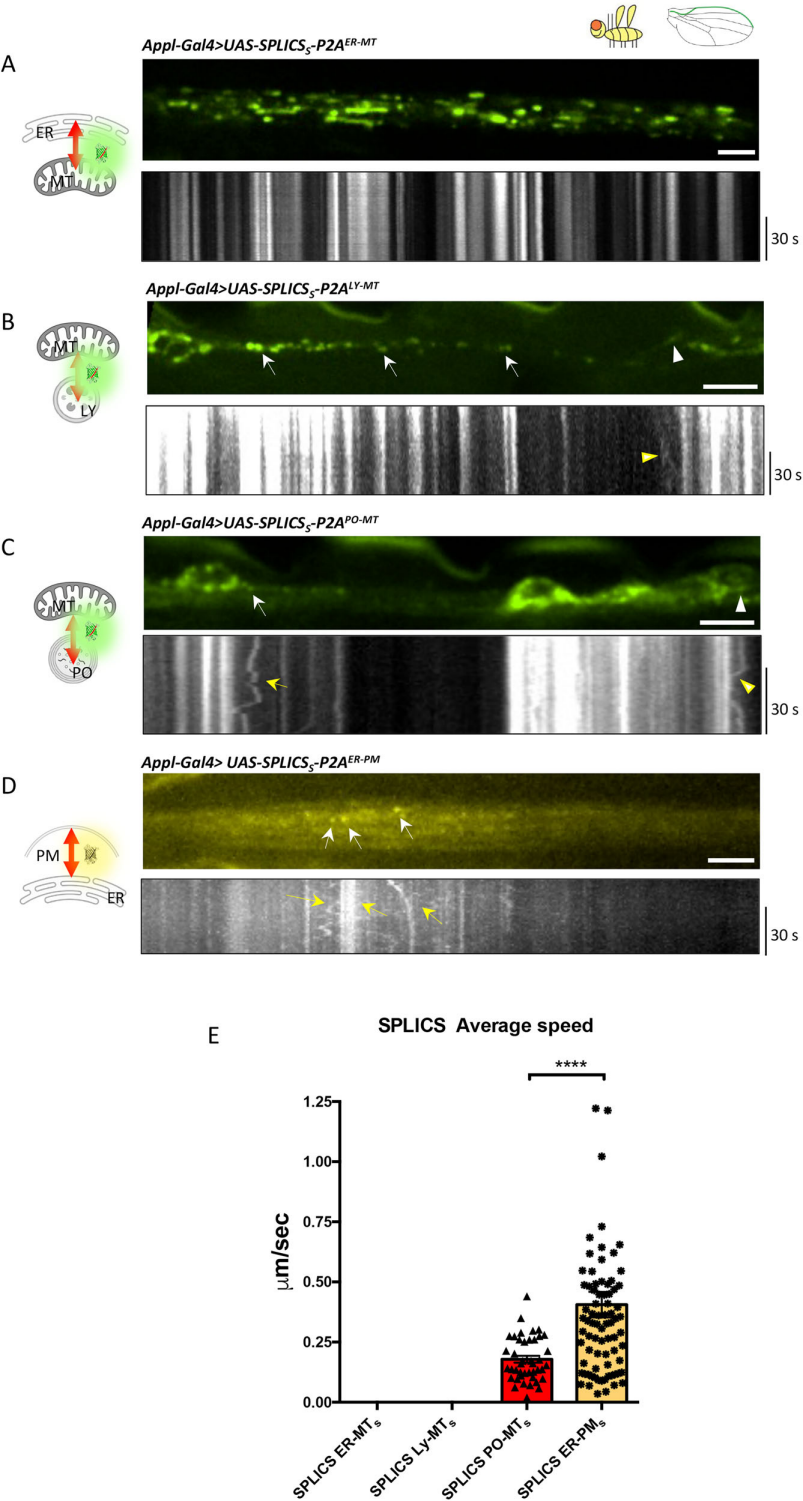
Live imaging experiments of SPLICS in transgenic *Drosophila melanogaster* wing neurons. Wing neurons expressing (**A**) *UAS*-*SPLICS*_*S*_*-P2A*^*ER-MT*^ (attP16), (**B**) *UAS-SPLICS*_*S*_*-P2A*^*LY-MT*^ (attP40), (**C**) *UAS-SPLICS*_*S*_*-P2A*^*PO-MT*^ (attP40), and (**D**) *UAS-SPLICS*_*S*_*-P2A*^*ER-PM*^ (attP40), were imaged by time-lapse spinning disk microscopy. Top panels in (**A**–**D**) are still images showing the wing nerve in the arch (**A**, **D**) and margin area (**B**, **C**). Bottom panels show kymographs from time series. White/yellow arrows and arrowheads indicate contact sites in the axons and cell bodies, respectively. **E** Quantification of average speed of SPLICS contact sites. N of moving dots= 42, 80. Statistical analysis was performed using Mann-Whitney’s test. ****P < 0.0001. Scale bars are 5 μm.

We next asked whether the characteristic of the dynamics of the different contact sites was conserved across different model organisms. To this end and to further validate the potential of the newly tested SPLICS probes, we extended our evaluation to *Drosophila melanogaster*. We and others have already established the functionality of the SPLICS system in this model organism by generating and validating transgenic fly lines expressing the SPLICS probe for ER-MT contact site detection [9, 10]. Now, we developed new transgenic fly lines for the *SPLICS*_*S*_*-P2A*^*LY-MT*^, *SPLICS*_*S*_*-P2A*^*PO-MT*^, and *SPLICS*_*S*_*-P2A*^*ER-PM*^ probes. By exploiting the UAS/Gal4 system, we specifically expressed these probes in the neurons of *Drosophila*. To analyze contact sites, we performed in vivo imaging of the sensory neurons of adult fly wings which, similarly to RB neurons, offer the possibility of performing concomitant imaging of axons and somal compartments from the same preparation [11, 12]. Co-localization of mitochondria was also checked for mitochondria-involving contacts (Supplementary Fig. 2). Representative images (Fig. 3A–D) show that in the wing neurons of 2-day-old flies, all SPLICS probes displayed the expected dotted pattern, confirming the in vivo applicability in flies. Additionally, the distinct distribution of these contacts suggests a specificity in the formation of different inter-organelle connections. Specifically, contacts marked by the ER-MT and ER-PM probes (inserted in the attP16 and attP40 landing sites, respectively) were equally evenly distributed throughout both the soma and axons of wing neurons. In contrast, LY-MT SPLICS (inserted in attP40) were enriched in the proximal tract of neuronal axons, rather than evenly marking the entire axonal bundle, while PO-MT contacts (also marked by SPLICS inserted in attP40) were primarily localized to the neuronal soma. This restricted distribution was independent of the specific insertion site as LY-MT and PO-MT probes inserted in attP2 displayed the same differences in their distribution (Supplementary Fig. 3). Due to the variable distribution of SPLICS, quantitative analysis could not rely on a fixed parameter but instead had to be adapted based on the specific probe pattern. For ER-MT contacts, we focused on the axonal bundle in the wing arch, which is rich in mitochondria, and quantified the number of contacts within a 50 µm region (Fig. 4A). For LY-MT contacts, we quantified the number of contacts within the first 20 µm of the axon, as the distribution of the probe made it possible to distinguish individual neurons in this region (Fig. 4B). For PO-MT (Fig. 4C) and ER-PM (Fig. 4D) contacts, we chose to quantify contacts in the neuronal soma, where the puncta appeared more distinct and easier to quantify, interestingly, the ER-PM contacts that were present in axons were often sparser and fainter than ER-MT contacts.

Next, we conducted time-lapse experiments to assess the dynamics of these contacts in vivo. Interestingly, overall motility was less readily evident in this system (Fig. 4A–D), potentially indicating a specific behavior of the contacts in mature neurons. ER-MT contact sites, remained static throughout the imaging period (Fig. 4A), possibly reflecting differences between the two model organisms and developmental stages analyzed. LY-MT contacts were also stably localized in neurons over time, in analogy with what observed in *Danio rerio* RB neurons, and dynamic events were only very rarely observed (Fig. 4B). Bouts of motion were clearly observed only with the ER-PM (Fig. 4D) and, more sporadically, with PO-MT contact sites, with speeds comparable to those observed in zebrafish (Fig. 4C) (*SPLICS*_*S*_*-P2A*^*ER-PM*^, speed = 0.4057 µm/sec, *N* = 42; *SPLICS*_*S*_*-P2A*^*PO-MT*^ speed = 0.1787 µm/sec, *N* = 80). Supplementary Movies S2A–D are the raw original images.

### Expression and characterization of SPLICS_S_-P2A^ER-MT^, SPLICS_S_-P2A^ER-PM^, SPLICS_S_-P2A^PO-MT^ and SPLICS_S_-P2A^LY-MT^ in NSC-34 cells differentiated into motor neurons

**Fig. 5:**
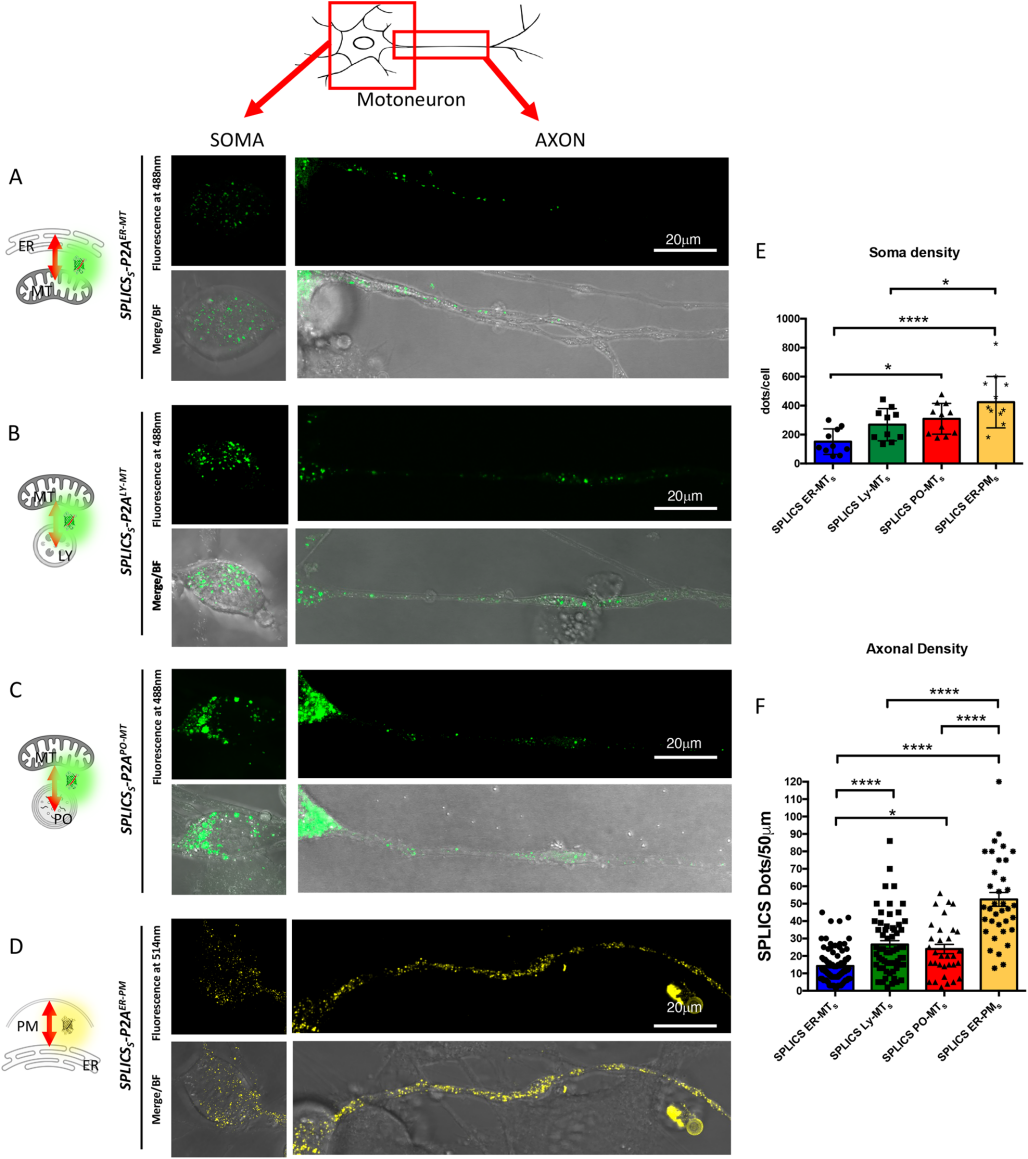
Expression of SPLICS in mouse motor neurons. NSC-34 cells differentiated into motor neurons expressing (**A**) *SPLICS*_*S*_*-P2A*^*ER-MT*^ (**B**) *SPLICS*_*S*_*-P2A*^*LY-MT*^ (**C**) *SPLICS*_*S*_*-P2A*^*PO-MT*^ and (**D**) *SPLICS*_*S*_*-P2A*^*ER-PM*^. The typical punctate pattern of SPLICS is observed in the soma and in the axons, BF is brightfield. Scale bar is 20 μm. **E** Density of SPLICS contact sites in the soma SPLICS/cell is quantified. N of cells= 10, 10, 11, 11. Statistical analysis was performed using one-way ANOVA and Tukey’s multiple comparisons. *P < 0.05, ****P < 0.0001. **F** Quantification of SPLICS axonal density as number of SPLICS in 50 μm. N of axons= 88, 61, 33, 36. Statistical analysis was performed using Kruskal-Wallis test with Dunn’s multiple comparison. *P < 0.05, ****P < 0.0001. Scale bar is 20 μm.

The data shown so far demonstrated that the mitochondrial partner organelle with whom a contact is established determines a specific signature in terms of dynamics and distribution, and that this dynamic is evolutionarily conserved being ER-PM > ER-MT = PO-MT > LY-MT. To confirm the relevance of the previously analyzed contacts in mammals, we selected the murine NSC-34 motoneuronal-like cell line as additional model. NSC-34 cells are readily differentiated in vitro and share several morphological and physiological characteristics with mature primary motoneurons [13, 14]. We transfected undifferentiated NSC-34 cells with the four SPLICS probes, targeting ER-MT, ER-PM, PO-MT, and LY-MT contacts, respectively. Twenty-four hours post-transfection, the culture medium was replaced with differentiation medium. After additional 48 h, the cells exhibited significant morphological changes, with about half of the population developing long protruding processes (Fig. 5A–D and Supplementary Fig. 4). At this stage, we conducted live imaging to investigate the behavior of the contacts within different cellular compartments. Co-localization of mitochondria was also checked for mitochondria-involving contacts (Supplementary Fig. 5). We observed a reconstituted fluorescent signal with a dotted pattern across all analyzed contacts (Fig. 5A–D). We quantified the number of contacts in the soma and in the first 50 µm of the axons of transfected cells (Mean ± SEM: soma-*SPLICS*_*S*_*-P2A*^*ER-MT*^ = 151.4 ± 28.03; *SPLICS*_*S*_*-P2A*^*ER-PM*^ = 423.5 ± 53.55; *SPLICS*_*S*_*-P2A*^*PO-MT*^ = 309.1 ± 32.02; *SPLICS*_*S*_*-P2A*^*LY-MT*^ = 268.5 ± 35.10; axon-*SPLICS*_*S*_*-P2A*^*ER-MT*^ = 14.22 ± 1.012; *SPLICS*_*S*_*-P2A*^*ER-PM*^ = 52.47 ± 3.963; *SPLICS*_*S*_*-P2A*^*PO-MT*^ = 23.97 ± 2.648; *SPLICS*_*S*_*-P2A*^*LY-MT*^ = 26.57 ± 2.212) being the density in the soma as follows: ER-PM > LY-MT = PO-MT > ER-MT (Fig. 5E). The axonal density among the different contacts was similar to that observed in zebrafish (Fig. 1E) with the exception of the ER-PM contacts that are much more abundant in NSC-34 cells (Fig. 5F).

### Time-lapse imaging of SPLICS_S_-P2A^ER-MT^, SPLICS_S_-P2A^ER-PM^, SPLICS_S_-P2A^PO-MT^ and SPLICS_S_-P2A^LY-MT^ in NSC-34 cells differentiated into motor neurons

**Fig. 6:**
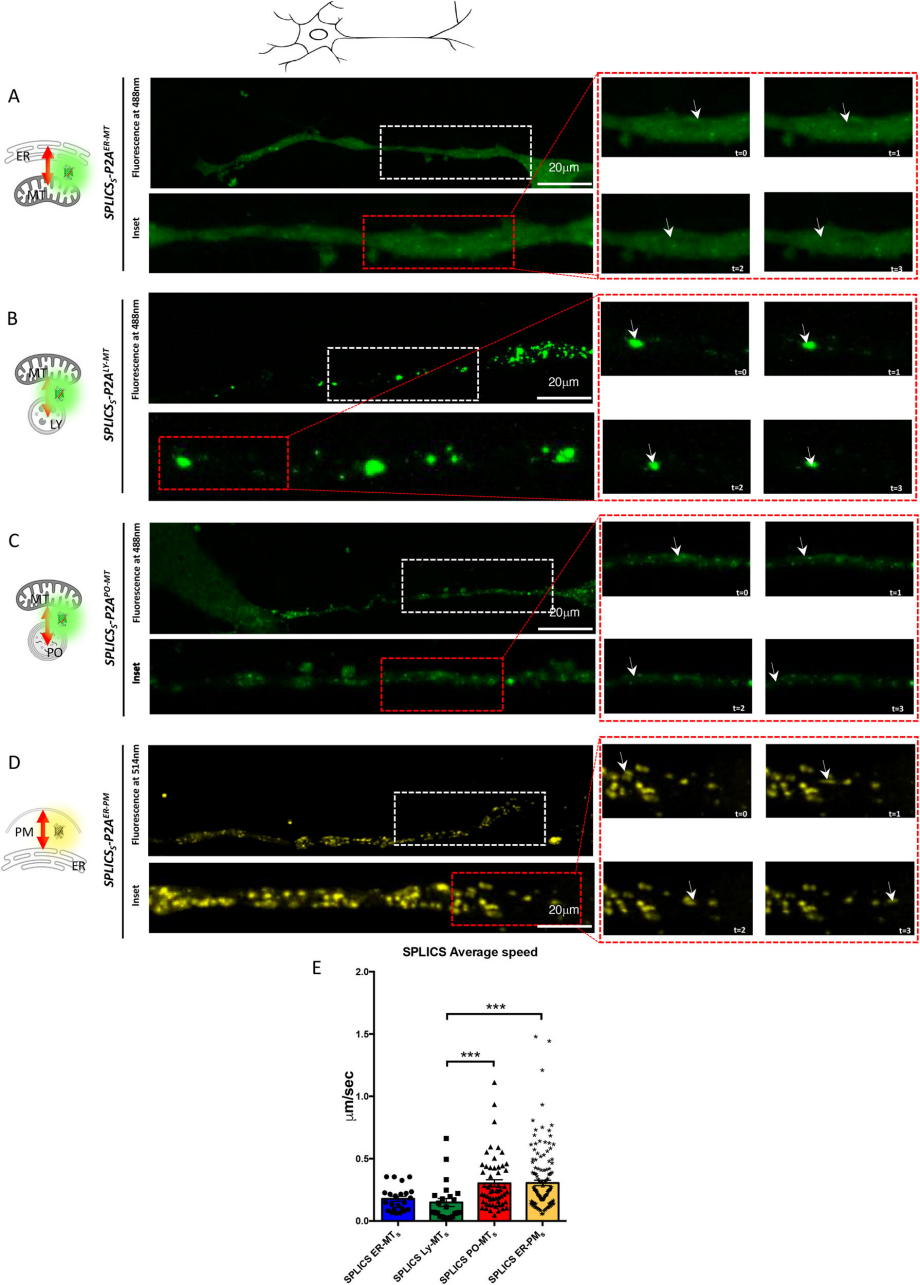
Time-lapse experiments of SPLICS in mouse motor neurons. Motor neurons expressing (**A**) *SPLICS*_*S*_*-P2A*^*ER-MT*^ (**B**) *SPLICS*_*S*_*-P2A*^*LY-MT*^ (**C**) *SPLICS*_*S*_*-P2A*^*PO-MT*^ and (**D**) *SPLICS*_*S*_*-P2A*^*ER-PM*^ were imaged over time. Frame-by frame images of selected time points are shown on the red ROI in the right panel (arrowhead indicates a specific contact site). White ROI is the inset. **E** Average speed of SPLICS contact sites in μm/sec is quantified. N of moving dots= 23, 24, 56, 129. Statistical analysis was performed using Kruskal–Wallis test with Dunn’s multiple comparison. ***P < 0.001. Scale bar is 20 μm.

Upon recording the axons of transfected cells, we observed distinct dynamics for the different contacts (Fig. 6A–D and right panels) (average speed: *SPLICS*_*S*_*-P2A*^*ER-MT*^ = 0.1779 ± 0.02034 µm/sec; *SPLICS*_*S*_*-P2A*^*ER-PM*^ = 0.3056 ± 0.02189 µm/sec; *SPLICS*_*S*_*-P2A*^*PO-MT*^ = 0.3026 ± 0.02851 µm/sec; *SPLICS*_*S*_*-P2A*^*LY-MT*^ = 0.1485 ± 0.03245 µm/sec). The quantification of the average speed revealed increased movement for ER-PM and PO-MT contacts compared to the less mobile LY-MT contacts. Supplementary Movies S3A–D are the raw original images. These findings are in line with the results shown above and suggest a contacts specificity in their mobile behaviors that is independent from contacts abundance, further reinforcing the data obtained in *Danio rerio* and *Drosophila melanogaster*.

## Discussion

Studying the dynamics of organelle contact sites has become an important aspect of intense research due to their crucial roles in cellular function. However, the ability to study these interactions in real-time and under physiological conditions remains a significant challenge, largely due to the current limitations in imaging technologies. These constraints have hindered our understanding of how these contact sites behave in vivo, particularly during rapid events that are difficult to capture. Despite the growing acceptance of the concept that organelle contacts are dynamic, involving continual assembly and disassembly, much less is known about the dynamic nature of these contacts once they are formed. Questions regarding the specificity of their duration and speed, and whether these characteristics are conserved across different species, remain largely unexplored. We started to fill these gaps by employing SPLICS reporters to observe the kinetics of the ER-MT interface in vivo over time, and this approach allowed us to gain novel insights into the behavior of these contacts [6]. Our initial observations revealed that long-range interactions undergo more extensive remodeling than short-range interactions, which are distinguished by a longer half-life, slower movement, and a higher probability of being stable over time. These findings suggest that not all organelle contacts are equally dynamic, and that their behavior may vary significantly depending on the nature of the interaction. To further investigate this phenomenon, we now selected four distinct organelle contact sites ER-MT, ER-PM, MT-PO, and LY-MT and used SPLICS reporters to monitor their dynamics in vivo across three different model systems: *Danio rerio*, *Drosophila melanogaster*, and mouse motor neuron cells. Our results revealed striking differences in contact sites dynamics. We showed variability in the dynamics of ER-MT and PO-MT contact sites, with ER-MT contacts demonstrating similar behavior in zebrafish and motoneurons but lacking dynamicity in fruit fly. In contrast, PO-MT contacts exhibited comparable kinetics in both zebrafish and fruit fly, though not in motoneurons. Meanwhile, LY-MT and ER-PM contact sites showed generally conserved dynamics across all the models analyzed. Notably, LY-MT contacts were less dynamic than the other contact types, suggesting a more stable interaction. In contrast, ER-PM contacts were the most dynamic, exhibiting the fastest kinetics in all models studied. This partial conservation may underscore the importance of these dynamics in maintaining cellular homeostasis, and any deviation from these basal dynamics could potentially lead to disease states. Thus, we propose that the dynamics of organelle contact sites represent a previously unrecognized parameter that should be carefully monitored in both health and disease contexts. The implications of our findings are significant, as they open new avenues for understanding how alterations in organelle contact dynamics might contribute to disease pathology. At the same time, our results generate new questions: we showed that the contacts can move by long-range directed transport, as in the case of ER-MT and ER-PM in zebrafish and Drosophila axons, but also through bidirectional, oscillatory-like behavior, as in the case of LY-MT and PO-MT in zebrafish axons and Drosophila cell bodies, respectively. The variety of dynamic behaviors observed with the ER-PM SPLICS is unexpected. The higher speeds of numerous long-range trafficking events recorded in the three systems presented in this study suggest the membranes marked by this specific reporter could be cargoes for motor-based cytoskeletal transport. On the face of it, this does not seem consistent with the more confined dynamics of ER-plasma membrane intersections expected in non-migrating cells. It will be important to study in future work the specificity of this interaction and whether they would be already functional on the transport vesicles or become functionalized once at destination. Whatever the case, this study highlights the need for a better understanding of the molecular machinery underlying the specific dynamic processes of contact sites formation and maintenance. We speculate that short- and long-range contact movement may reflect different cellular functions. Future work will establish the mechanisms underpinning the specific dynamic behavior of contact sites. One important open question is whether the movement is retrograde or anterograde: as for the Zebrafish experiments this is not easily established since bundles of neuronal projections from different isolated neurons are overlapping. In *Drosophila* and motoneuron cells, however, both the directions are often covered suggesting that bidirectional movement can be achieved. Additional experiments are needed to specifically solve this point. Whether different contacts are only moved in specific direction is also unknown. How are these interactions regulated at the cellular level and at different developmental stages? How is coordination achieved between different contact sites within a single cell, ensuring that multiple organelle interactions are synchronized? These questions highlight the need for further experimental investigations to unravel the complex regulatory networks that govern organelle contact dynamics. Understanding these processes in greater detail could provide new insights into the mechanisms of cellular organization and the development of novel therapeutic strategies for diseases associated with organelle dysfunction.

## Methods

### Zebrafish husbandry and transgenic lines

All animal experiments were conducted on wild-type fish. Adult fish were maintained and raised in 5 l tanks with freshwater at 28 °C with a 12 h light/12 h dark cycle. Embryos were obtained from spontaneous spawnings and raised at 28 °C in Petri dishes containing fish water45. To perform experiments, both wt and *s1102t:GAL4* fish were used. All experiments were conducted on 24 h post fertilization (hpf) embryos.

### Zebrafish imaging

The pT2-DsRed-UAS-SPLICSS-P2A vector has been already described. Before injections, all plasmids were diluted in Danieau solution (58 mM NaCl, 0.7 mM KCl, 0.4 mM MgSO4, 0.6 mM Ca(NO3)2, 5 mM HEPES pH 7.6) and 0.5% phenol red. At 24 hpf, embryos were screened for fluorescence, dechorionated, and anesthetised with tricaine. They were then mounted on 8-well chambers (square of well 1 cm^2^, ibidi-80806) in low melting agarose (1.3%, EuroClone). Fish water containing tricaine methanesulfonate 0.61 mM (Sigma) was added in each well, in order to keep fish anesthetised. Mounted fish were imaged at RT (20–23 °C) using a Leica TSC SP5 inverted confocal microscope, using a HCX PL APO 63X/numerical aperture 1.40–0.60 oil-immersion objective. A Z-stack of every cell was acquired. Representative time-lapse recordings were acquired with a frame interval of 3–6 s for a total time of 2 min. Time-lapse movies were analyzed with Fiji in order to obtain kymographs.

### Drosophila husbandry and generation of transgenic flies

Flies were maintained on “Iberian” food [70 mg/ml yeast (Brewer’s yeast, MP Biomedicals, 903312), 55 mg/ml glucose (VWR, 10117HV), 7.7 mg/ml agar (SLS, FLY1020), 35 mg/ml organic plain white flour (Doves Farm, UK), 1.2 mg/ml Nipagin (Sigma, H3647), 0.4% propionic acid (Sigma-Aldrich, P5561] at 25 °C and 60% humidity with a 12-hour light–12-hour dark cycle. The transgenic fly lines generated in this study were obtained by phiC31-mediated transgenesis to integrate the relevant constructs into either the attP40 (25C6) or attP2 (68A4) landing sites following embryo injection.

### Drosophila imaging

Imaging was performed as previously described [15]. Briefly, 2 days-old flies that had been anaesthetised with CO_2_ were immobilised, with wings outstretched, on a cover glass with a fine layer of Voltalef 10S halocarbon oil (VWR). A second coverglass was then added on top of the fly to stabilize the sample. Wing nerves were imaged by spinning disk microscopy at 0.5 frames per seconds to acquire time-lapse video. Image series were captured for 1-2 min. Tracking of SPLICS was performed manually (MTrackJ) on the raw movies by marking the start and end of each run and velocities calculated using the Velocity Measurement Tool (http://dev.mri.cnrs.fr/projects/imagejmacros/wiki/Velocity_Measurement_Tool). Kymographs were generated from time series using the Multi Kymograph tool in Fiji/ImageJ. SPLICS were quantified by counting clearly discernable puncta in single focal planes. In cases where the sample shifted during filming, correction was applied with the StackReg plugin of ImageJ.

### NSC-34 cell line

The mouse motor neuron-like hybrid cell line NSC-34 was maintained in DMEM high glucose (gibco 41966-029) containing 10% FBS (Sigma) and 1% penicillin/streptomycin (Euroclone ECB3001D) in a humidified atmosphere containing 5% CO_2_ at 37 °C. For the experiments, 18000 cells/well were seeded in 8-well chambers (square of well 1 cm^2^, ibidi-80806) coated with collagen (50 μg/ml in acetic acid 0.02 M). The day after, each well was transfected with a SPLICS reporter (0,5 μg DNA/well for ER-PM, PO-MT, LY-MT, -while 0,35 μg DNA/well for ER-MT) using 1,25 μl/well of Lipofectamine 2000 (Invitrogen) for 6 h in 300 μl OPTI-MEM (gibco 31985-070). The medium was then replaced with DMEM high glucose containing 10% FBS and no antibiotics. The day after, to induce neurite outgrowth, medium was replaced with DMEM F12 (gibco 11330-032) containing 1% FBS, 1% penicillin/streptomycin, 5 μM retinoic acid, 1X MEM-non-essential amino acids (gibco 11140-035) for 48 h. The medium was changed every day. After 48 h, cells were imaged using a confocal microscope Leica SP5, using a HCX PL APO 63X/numerical aperture 1.40–0.60 oil-immersion objective. Z-stacks of both cell somas and axons were acquired, with a step size of 0.5 μm. Time-lapse recordings of axons were acquired with a frame interval of less than 6 s for 2 min. The movements of particles were analyzed with imagej Fiji using the plugin MTrackJ.

### Statistical analysis

Results are reported as means ± SEM and Gaussian distribution was assessed by Shapiro-Wilk normality tests. Statistical analysis of two groups was obtained applying unpaired Student’s two-tailed t test. To compare more than two groups one-way/two-way ANOVA test was used. All statistical analyses were performed using GraphPad Prism version 8.00 for Mac OS X (La Jolla, California, USA). Statistical significance threshold was set at *p* < 0.05. *p ≤ 0.05, **p ≤ 0.01, ***p ≤ 0.001, ****p ≤ 0.0001.

## Supplementary information

Supplementary Figures

Supplementary Movie S1A

Supplementary Movie S1B

Supplementary Movie S1C

Supplementary Movie S1D

Supplementary Movie S2A

Supplementary Movie S2B

Supplementary Movie S2C

Supplementary Movie S2D

Supplementary Movie S3A

Supplementary Movie S3B

Supplementary Movie S3C

Supplementary Movie S3D

## Data Availability

All data and video are contained in the supplement. Further data are available are upon request.
