# Supplementary material for: Tracking spatiotemporal distribution of organelle contacts in vivo with SPLICS reporters
Source: Cell Death Dis. 2025 Mar 27;16(1):214. doi: 10.1038/s41419-025-07511-5 (PMC11950385; doi:10.1038/s41419-025-07511-5)
Supplement: Supplementary file 1 — Supplementary Figures [file 41419_2025_7511_MOESM1_ESM.docx]

**Supplementary Figures**

**Tracking spatiotemporal distribution of organelle contacts *in vivo* with SPLICS reporters.**

*Lucia Barazzuol^1^, Tetiana Tykhonenko^1^, Tia L. Griffiths^2^, Alessio Vagnoni^2,3*^, Marisa Brini^4,5 *^ and Tito Calì^1,,5,6 *^*

**
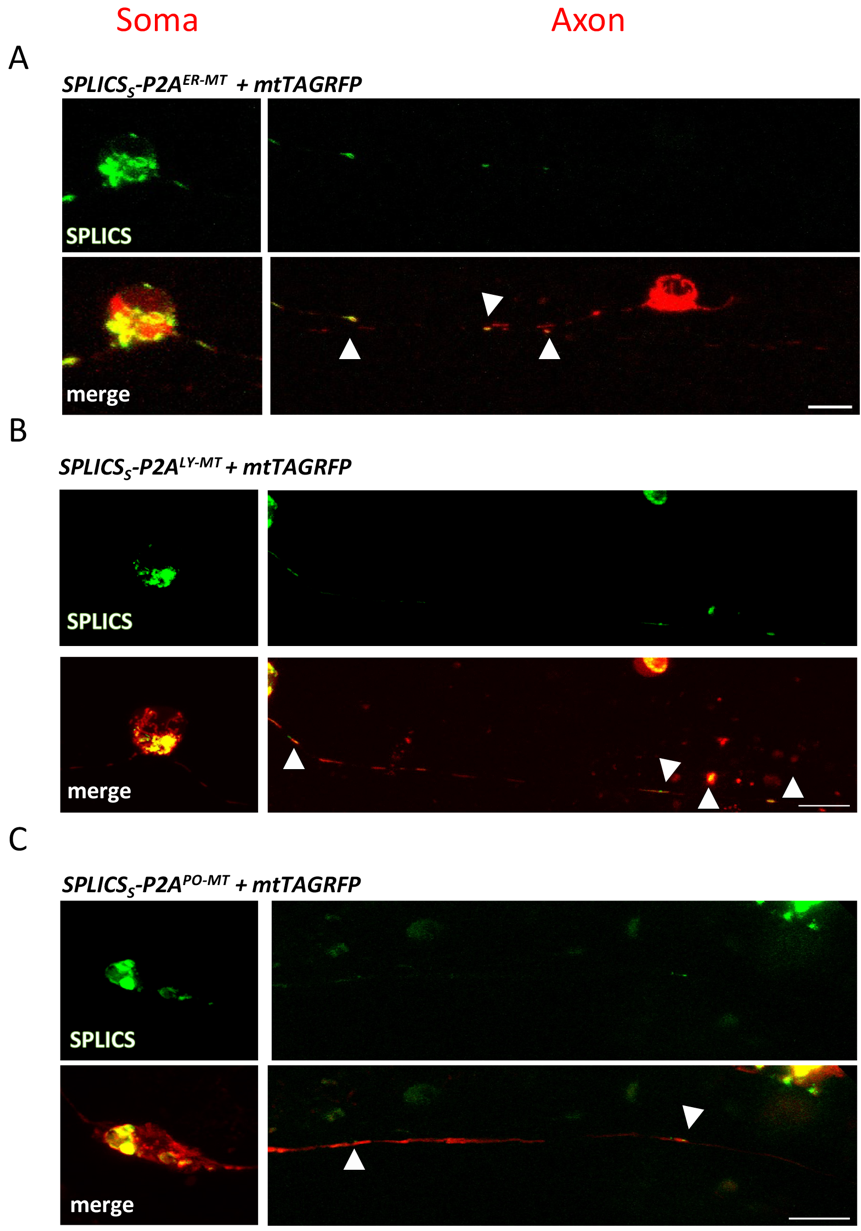
**

**Supplementary Figure 1. SPLICS colocalization with the mitochondria in Danio Rerio neurons**

Rohon Beard neurons expressing mitochondria-targeted TAGRFP with (A) *SPLICS_S_-P2A^ER-MT^*, (B) *SPLICS_S_-P2A^LY-MT^*, and (C) *SPLICS_S_-P2A^PO-MT^*. Pseudo-coloured green and red images show SPLICS and mitochondria, respectively. Discrete clusters of SPLICS puncta colocalize with the mitochondria in the soma and more defined ones in the axons (arrows) of neurons. Scale bars: 10 μm.

**
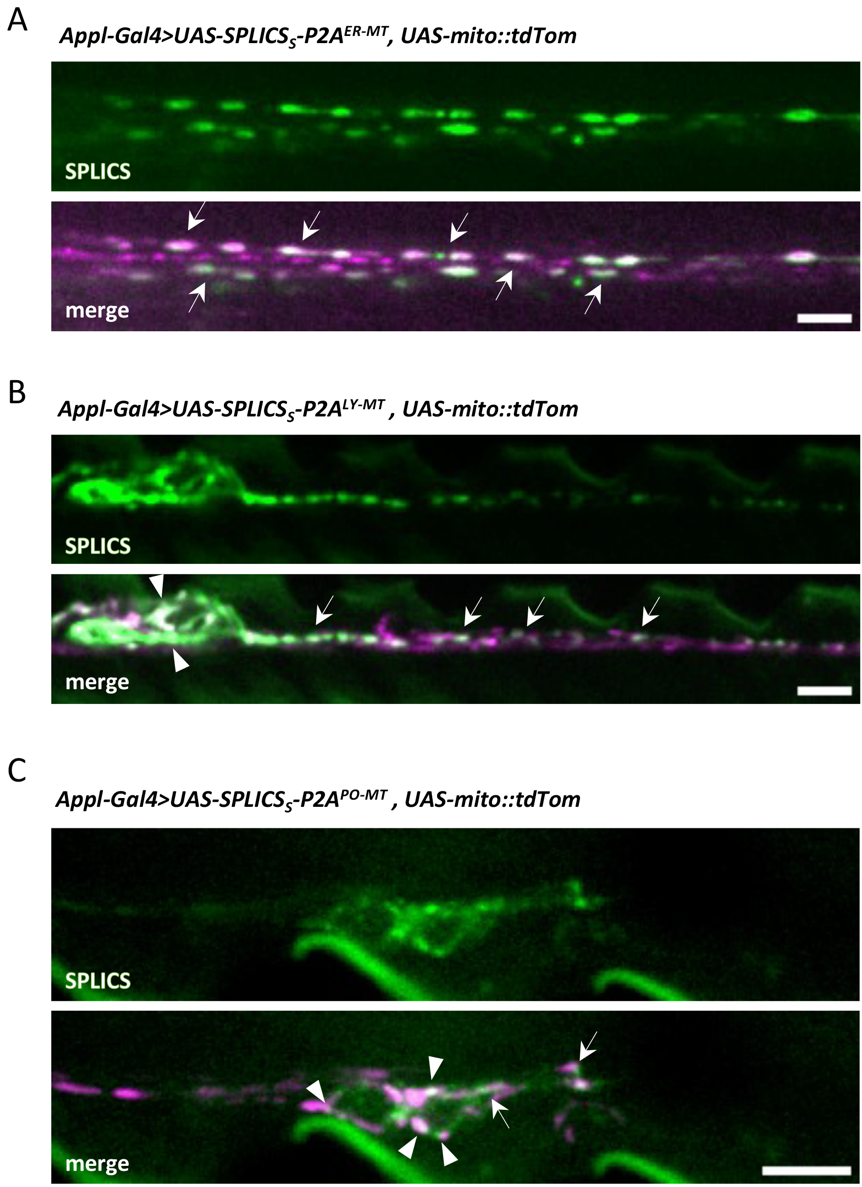
**

**Supplementary Figure 2. SPLICS colocalization with the mitochondria in *Drosophila* neurons**

Wing neurons expressing mitochondria-targeted tandem Tomato (*UAS-mito::tdTom)* (12) with (A) *UAS-SPLICS_S_-P2A^ER-MT^*, (B) *UAS-SPLICS_S_-P2A^LY-MT^*, and (C) *UAS-SPLICS_S_-P2A^PO-MT^*. Green and magenta pseudo-colours mark SPLICS and mitochondria, respectively. Discrete clusters of SPLICS puncta colocalize with the mitochondria in the soma (arrowheads) and the axons (arrows) of neurons. Scale bars: 5 μm.

**
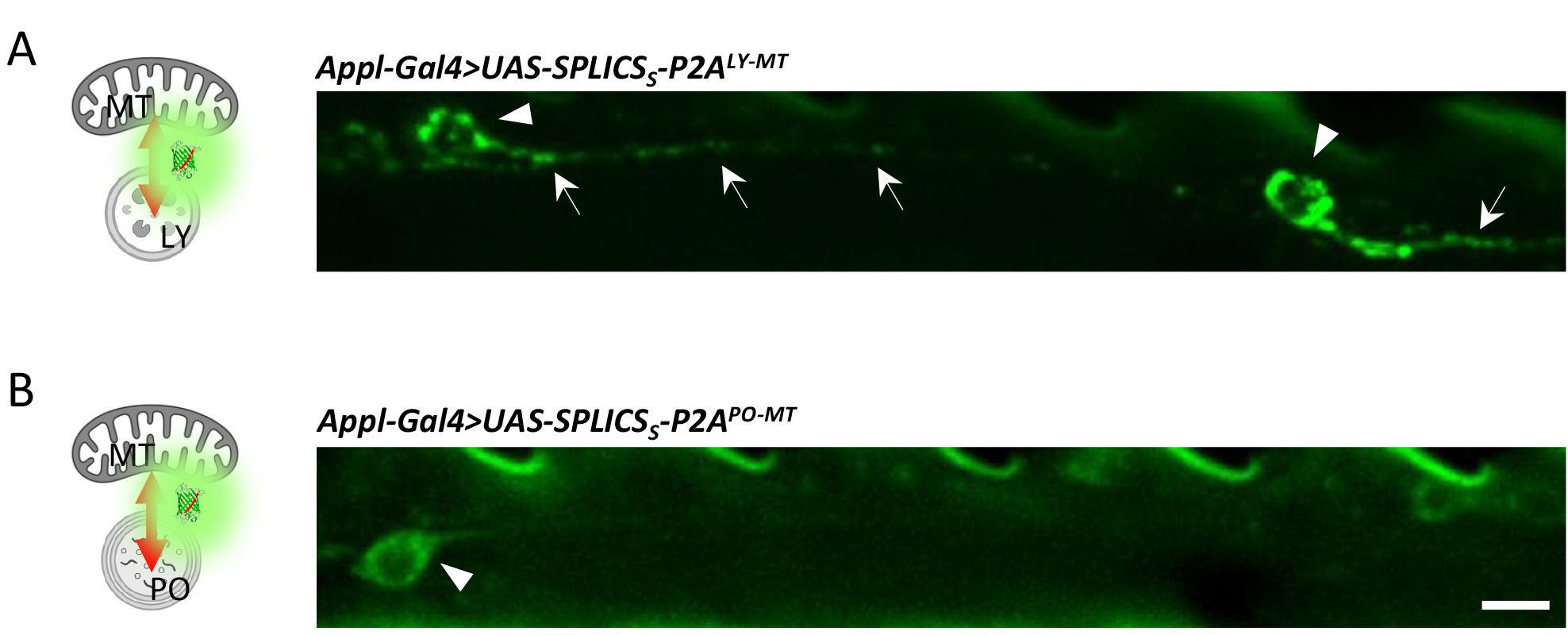
**

**Supplementary Figure 3 Expression of SPLICS in transgenic *Drosophila melanogaster* wing neurons.** Wing neurons expressing A) *UAS-SPLICS_S_-P2A^LY-MT^* (attP2) and B) *UAS-SPLICS_S_-P2A^PO-MT^* (attP2) in the cell bodies and axons of the Drosophila wing. The distribution of the probes inserted in the attP2 landing site mirrors the pattern observed with the attP40 insertions (Fig. 3-4). Arrows and arrowheads indicate contacts in the axons and cell bodies, respectively. Scale bar is 5μm.


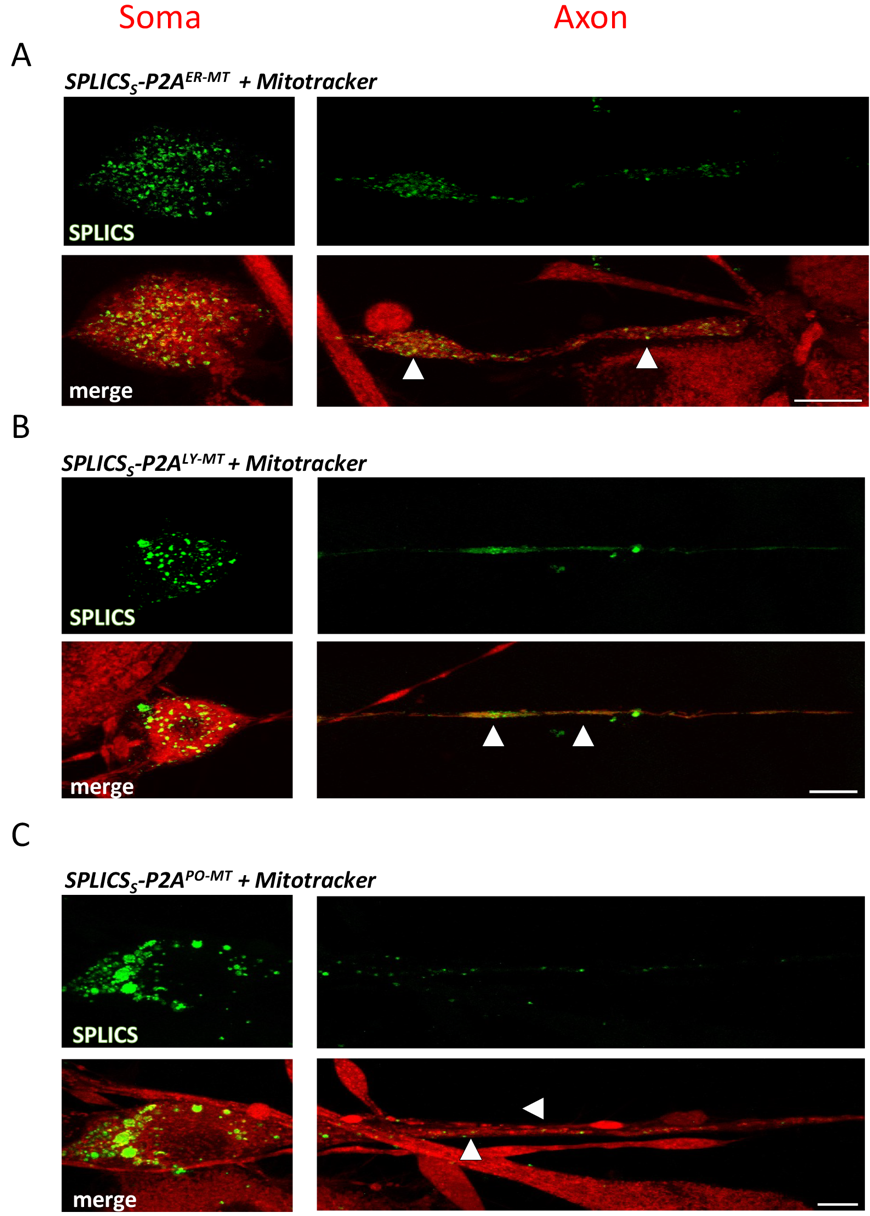


**Supplementary Figure 4. SPLICS colocalization with the mitochondria in NSC-34 motoneurons**

Differentiated motoneurons incubated with MitoTracker™ Red CMXRos and expressing (A) *SPLICS_S_-P2A^ER-MT^*, (B) *SPLICS_S_-P2A^LY-MT^*, and (C) *SPLICS_S_-P2A^PO-MT^*. Green and red pseudo-colours mark SPLICS and mitochondria, respectively. SPLICS puncta colocalize with the mitochondria in the soma and the axons of neurons. Scale bars: 10 μm.


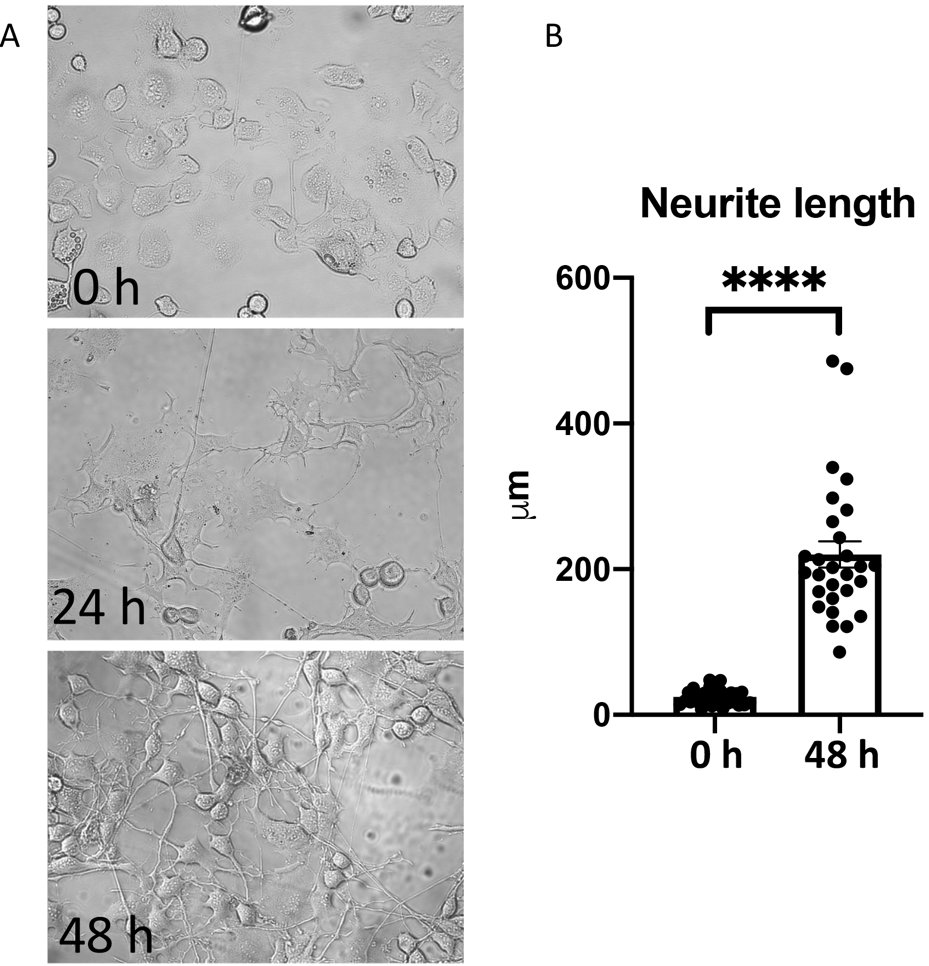


**Supplementary Figure 5. Differentiation of NSC-34 cells**

(A) Representative images of NSC-34 cells: undifferentiated (0 h), displaying short neurites, and upon 24 or 48 h incubation in the differentiation medium, exhibiting a progressive outgrowth of neurite projections. Images were obtained at 20X magnification using an inverted epifluorescence microscope. (B) Morphological differentiation of NSC-34 cells determined by measurement of neurite length in undifferentiated cells and after 48 h of differentiation. N of axons= 28, 28. Statistical analysis was performed using Mann-Whitney test. ****P< 0.0001.
